# Stress among Croatian physicians: comparison between physicians working in emergency medical service and health centers – pilot study

**DOI:** 10.3325/cmj.2011.52.8

**Published:** 2011-02

**Authors:** Ljiljana Gregov, Ana Kovačević, Ana Slišković

**Affiliations:** Department of Psychology, University in Zadar, Zadar, Croatia

## Abstract

**Aim:**

To determine the sources of stress, its intensity, frequency, and psychophysical and behavioral reactions in physicians working in emergency medical service and those working in health centers.

**Methods:**

To a convenience sample of primary care physicians employed in emergency medical service (n = 79) and health centers (n = 81), we administered the list of demographic questions, Scale of Sources of Stress, Scale of Intentions of Leaving the Job, and Scale of Psychosomatic Symptoms.

**Results:**

Emergency medicine physicians experienced significantly more intense and more frequent uncontrollable working situations, conflict between work and family roles, and unfavorable relationships with coworkers than physicians working in health centers. They were also more likely to leave the job during the next few years and/or change jobs within the profession (scores 2.2 ± 0.9 vs 1.7 ± 0.9 out of maximum 5.0, F = 12.2, *P* = 0.001) and they had a poorer physical health status (scores 1.8 ± 0.5 vs 1.7 ± 0.5 out of maximum 4.0, F = 5.3, *P* = 0.023).

**Conclusion:**

Physicians working in emergency medical service experience more stress in almost all aspects of their work than physicians working in health centers. They also have a stronger intention of leaving the job, which decreases with years of experience.

It has been shown that health workers are highly susceptible to stress at work and experience more negative outcomes of stress than other professions (1-8). For example, a survey conducted in Irish hospitals indicated that work stress caused dissatisfaction in 79% of physicians, 56% evaluated their job as stressful or extremely stressful, and as many as 68% considered leaving the profession, half of them very seriously (9). Work stress in physicians is associated with an array of other negative outcomes: adverse psychological well-being (6), job burnout (10), significantly larger number of suicide attempts (11,12), alcohol dependence, and other psychosocial problems (11). Stress in physicians affects not only their private lives and health but also the quality of medical care that they provide, patient’s satisfaction with the physician, and patient’s adherence to treatment (13).

Some of the most important sources of stress for general practitioners and hospital physicians of different specializations were identified as intrinsic factors of work, administration, stressors related to financial opportunities, contact with patients, relationships with coworkers, organizational structure and climate, and interference of private and work life (4-6). One of the most stressful areas of medicine is emergency medicine, in which physicians and other medical personnel are frequently exposed to unexpected and serious medical conditions, trauma, and life or death situations. Important sources of stress specific to work in emergency medicine were found to be time pressure and the need to make critical decisions (8), with numerous negative effects of stress, particularly the intention of leaving the job and deteriorated psychological well-being (11,14-16).

The activities of emergency medical service in Croatia include the treatment of severe acute and urgent conditions at the site of the incident, at the dispensary in which the patient arrived, and during transport to the nearest hospital or other health care institution (17,18).

The aim of this pilot case study was to identify specific sources of stress in emergency medicine physicians in Croatia, since we assumed that, due to the unpredictable nature of their job, they have different perception of sources of stress, health status, and intent of leaving the job than physicians working in health care centers.

## Methods

### Participants

We sent official requests for research to the 4 emergency medical facilities in Croatia – Zagreb, Split, Osijek, and Rijeka, and to 4 health centers (Zagreb-West, Split-Dalmatia, and Istria). We received approval from the following institutions: emergency medical services in Zagreb and Split and Health Center Zagreb-West, Health Centre Split-Dalmatia, and health centers in Istria. According to the approximate number of employees in these institutions, a total of 360 questionnaires were sent.

Of the total of 160 physicians who answered the questionnaire, 79 were general practitioners working in emergency medical institutions and 81 general practitioners working in health centers. Physicians working in health centers were older (48.3 ± 10.2 vs 42 ± 10.1 years, *t* = 3.9, *P* < 0.001), had more total years of working experience (22 ± 11 vs 15.6 ± 10 years, *t* = 3.8, *P* < 0.001), and more years of service at the current workplace (15.1 ± 11.5 vs 11.6 ± 9.9 years, *t* = 2.0, *P* = 0.047) than physicians working in emergency medical institutions. This is probably because emergency medical services in Croatia employ mostly young physicians, most of whom leave this branch of medicine after the internship. The total sample included 123 women and 37 men. The two groups did not differ in sex, marital status, and the number of children.

### Procedure

After the research had been approved by the respective hospital directors, the questionnaires were mailed to one employee of the hospital assisting in the research who forwarded them to the physicians. To ensure anonymity of the data, each questionnaire was placed in a separate envelope, together with an explanation of the purpose of research and detailed instructions about the completion. The completion took approximately 15 minutes.

### Measures

The following measuring instruments were used: a list of demographic questions, the Sources of Stress Scale, Questionnaire About the Intentions of Leaving the Job, and Scale of Psychosomatic Symptoms.

The list of sociodemographic questions collected data on sex, age, total work experience, years of experience at the present job, marital status, and children.

The Sources of Stress Scale consists of a total of 60 items, ie, 5 subscales on sources of stress (additional web material, Appendix 1)(web extra material 1). The subscales were constructed on the basis of the measuring instruments used in studies on stress in physicians and medical staff in emergency medicine and other specializations (5,15,19), and in collaboration with physicians working in the Emergency Medical Service in Zadar. Exploratory factor analysis confirmed the one-dimensionality of each of the 5 subscales, with each measure having satisfactory internal reliability (not shown).

The frequency of individual stressors was determined on a 3-degree scale (never/occasionally/frequently) and the intensity on a 4-degree scale (not at all/not generally/mostly/very stressful). The sources of stress were the following:

1. “Uncontrollable work situation,” which consists of 17 items and has the reliability of internal consistency (Cronbach α) of 0.917. Uncontrollable work situations include the anticipation of unexpected and urgent intervention, attendance and expectation of a call for help, the unpredictability of work, night visits and interventions, fear of infection with a contagious disease, conducting emergency response at inappropriate places and in inadequate conditions, the responsibility for patients' lives, lack of time for punctual intervention, fear of the media and legal prosecution, course and timing of treatment, coping with atypical symptoms of known diseases, the ability to provide adequate treatment, the patient coping with progressive illness and poor prognosis, the lack of relevant data for making proper decisions about diagnosis and treatment, or situations where the main characteristic is a lack of control.

2. “The organization of work and working conditions,” which consists of 14 items and the content of which refers to adherence to prescribed financial recommendations of the ministry of health, time pressure and lack of time to perform the entire job, poor organization of work when there are a lot of patients in the waiting room, a sense of decreased working efficiency, performing administrative tasks, finding a replacement when having some other obligations, a great amount of responsibility, impact of work on the quality of personal and family life, coping with a rare and little-known disease, a patient's refusal of the proposed methods of treatment, and a lack of the necessary work equipment and materials. Cronbach α for this source of stress was 0.870 (the details of validation of instruments are not shown).

3. “Conflict of work and family roles,” which consists of 10 items and has the reliability (Cronbach α) of 0.837. The content of the items refers to the impact of working hours on family/personal life, the impact of shift work on family/personal life, professional isolation, lack of recognition for contributions to the profession and work, lack of time for research, lack of sleep, the effect of work stress in communicating with relatives and friends, and division of time between private and professional obligations.

4. “Unfavorable relationships with colleagues,” which consists of 11 items and has the reliability (Cronbach α) of 0.830. The content of the items refers to individuals making the right decisions, problems in relationships with colleague physicians, conflict between their department and other health services, problems with the managers, fear of their own bad decisions and reactions, the coordination of decisions within the team, and conflicts with the junior/senior fellow physicians.

5. “Interaction with patients,” which consists of 8 items and the content of which relates to the inappropriate demands of patients, coping with unrealistically high demands of patients, coping with the problems of patients that are not directly associated with the disease, conflicts with problematic patients, and problems with premature discharge from hospital. Its reliability (Cronbach α) was 0.858.

The Questionnaire About the Intentions of Leaving the Job consists of 4 items evaluated on a scale from 1 – no intention of leaving the job to 5 – full intention of leaving the job (additional web material, Appendix 2)(web extra material 2). The items refer to the intention of leaving the job during the next few years, the intention of changing the job within the profession, intention of leaving the direct patient care, and the intention of completely leaving medical work. This questionnaire was designed for research on physicians and contained items exclusively related to the profession. Similar versions of this questionnaire were used in studies on physicians, nurses, and other health staff (14). Factor analysis produced only one factor, which explained 47.344% of the variance. Further analysis included all of the items because it had satisfactory psychometric properties. Internal consistency (Cronbach α) of this type of survey is 0.735 and all items have a higher than 0.50 correlation with the total score.

The Scale of Psychosomatic Symptoms was constructed for this research. It consists of 13 items, which are assessed on a scale from 1 – “never” to 4 – “always” (additional web material, Appendix 3)(web extra material 3). The participants rated how often they felt some disturbances in the past year, such as: pain in their feet, insomnia, drowsiness, breathing difficulty, swollen joints, and sore muscles, feelings of fatigue in the morning, headaches, pressure or chest pain, rapid or irregular heartbeat, gastrointestinal disturbances, high blood pressure, poor appetite, and the feeling of fatigue and exhaustion. The scale of psychosomatic symptoms revealed the existence of two latent factors that explained 39.4% of the variance. Factors were “physical health” and “psychological health.” The item “high blood pressure” was excluded from further analysis, because it showed unsatisfactory psychometric properties (low correlation with the total score, low saturation on all factors; its removal increased internal consistency of the questionnaire and the Cronbach α). The Cronbach α of the subscale “physical health” was 0.754 and the subscale of “psychological health” 0.757.

### Statistics

For data processing, we used STATISTICA, version 8 (StatSoft, Tulsa, OK, USA). Differences between groups of physicians were assessed using *t*-tests and analysis of variance and covariance.

Exploratory factor analysis, common factor method with the Gutman Kaiser criterion for factor extraction (typical root >1), and Varimax rotation were used in the analysis of the factor structure of individual measurement scales. For the factor analysis, we calculated the index of stressfulness of sources of stress by multiplying the frequency and intensity of stress. This makes it possible to see which stressors were a real threat, with a higher index indicating greater intensity of stressors which frequently occur. However, before calculating the product of frequency and intensity, we added a constant value 0.5 to the scale of frequency. In this way it is possible to distinguish the index of stressfulness of the situations in which the stressors that occur sometimes are mainly stressful (1.5 × 2) and the situations in which the stressors that occur often are mainly not stressful (2.5 × 1). Namely, stressful work situations, even if do not occur on daily basis, have greater stressfulness than usual work situations which are not perceived as stressful.

The reliability of internal consistency was determined using Cronbach α coefficient. The correlations among variables were tested using Pearson correlation coefficient. Due to multicollinearity of sources of stress (high correlations between them), multiple regression analysis with stress outcome measures as criteria variables was not applied. Multicollinearity in this case poses difficulties in testing and interpreting regression coefficients. Therefore, bivariate correlations between sources of stress and stress outcome measures are given for the two samples of physicians.

## Results

The response rate to the survey was 44.4%, as 160 out of 369 physicians returned the questionnaire.

### Perceptions of sources of stress in two groups of physicians

In order to compare the results on different stressors scales, the average values were calculated (total scale score divided by the number of items on scale).

Emergency medicine physicians experienced significantly more intense and frequent uncontrollable working situations, conflict between work and family roles, and unfavorable relationships with coworkers than physicians working in health centers (Table 1).

**Table 1:** Assessments of the stress sources, physical health, mental health, and intention of leaving the job in a convenience sample of emergency medical service physicians (EMCI) and general practitioners working at health centers (HC)

Investigated parameters	Theoretical score range	Observed score range (mean ± standard deviation) in		ANOVA (F)	ANCOVA (F)
		EMCI (n = 79)	HC† (n = 81)		
Uncontrollable work situations:					
frequency	0.0-2.0	1.4 ± 0.3	1.0 ± 0.3	57.1; *P* < 0.001	48.0; *P* < 0.001
intensity	0.0-3.0	1.9 ± 0.6	1.6 ± 0.6	14.2; *P* < 0.001	13.6; *P* < 0.001
Working conditions:					
frequency	0.0-2.0	1.2 ± 0.3	1.3 ± 0.4	1.4; *P* = 0.236	0.8;*P* = 0.362
intensity	0.0-3.0	1.6 ± 0.5	1.8 ± 0.6	3.8; *P* = 0.052	3.8;*P* = 0.054
Work/family conflict:					
frequency	0.0-2.0	1.2 ± 0.4	0.9 ± 0.4	16.0; *P* < 0.001	13.7; *P* < 0.001
intensity	0.0-3.0	1.6 ± 0.6	1.4 ± 0.6	5.2; *P* = 0.024	5.0; *P* = 0.027
Unfavorable relationships with coworkers:					
frequency	0.0-2.0	1.1 ± 0.3	0.8 ± 0.3	23.2; *P* < 0.001	25.3; *P* < 0.001
intensity	0.0-3.0	1.5 ± 0.6	1.2 ± 0.6	11.1; *P* = 0.001	13.8; *P* < 0.001
Interaction with patients:					
frequency	0.0-2.0	1.2 ± 0.3	1.0 ± 0.4	9.1; *P* = 0.003	8.6; *P* = 0.004
intensity	0.0-3.0	1.7 ± 0.6	1.6 ± 0.6	1.3; *P* = 0.265	1.4; *P* = 0.245
Physical health	1.0-4.0	1.8 ± 0.5	1.7 ± 0.5	1.0; *P* = 0.330	5.7; *P* = 0.019
Mental health	1.0-4.0	2.4 ± 0.5	2.2 ± 0.5	3.3; *P* = 0.069	3.0; *P* = 0.087
Intention to leave job	1.0-5.0	2.2 ± 0.9	1.7 ± 0.9	12.2; *P* = 0.001	8.4; *P* = 0.004

To obtain detailed insight into the differences in the perception of important stressors, analysis of variance was performed specifically for the frequency and intensity of certain aspects of the job for all sources of stress. Physicians in emergency medical services were significantly more frequently exposed to all the examined stressors, except for work organization and working conditions (Table 1). Also, physicians in emergency medical service experienced uncontrollable working situations, conflict of work and family roles, and unfavorable relationships with colleagues as significantly more stressful than physicians working in health centers (Table 1).

### Health status and intention to leave jobs

Although analysis of variance did not show difference in health status between the two groups of physicians (Table 1), analysis of covariance found such difference (F = 5.3, *P* = 0.023) when the data were adjusted for age: physicians working in emergency medicine had significantly poorer physical health than physicians working in health centers.

**Figure 1:**
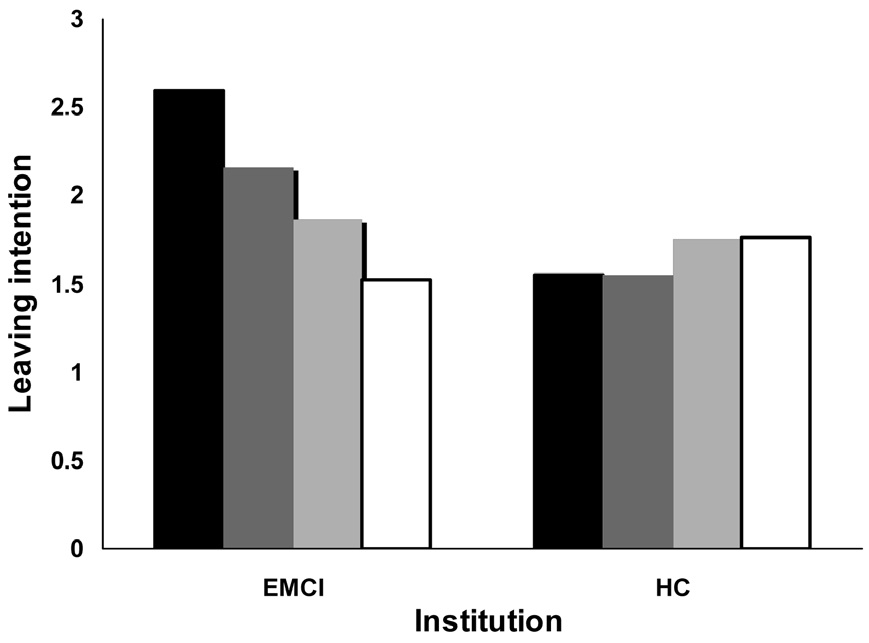
Intention of leaving the job according to years of working experience and institution in a convenience sample of emergency medical service physicians (EMCI) and general practitioners working in health centers (HC). Open columns – 0-9 years of work experience; closed columns – 10-19 years of work experience; light gray columns – 20-29 years of work experiences; dark gray columns – 30-40 years of work experience. F (institution) = 5.14, *P* = 0.025; F (years of working experience) = 1.37, *P* = 0.255; F (institution* years of working experience) = 3.15, *P* = 0.027.

Physicians working in emergency medicine had a greater intention of leaving the job than physicians working in health centers (Table 1). In order to determine the items in which the two groups differed, the analysis of variance was performed for each item in the questionnaire. It was found that physicians working in emergency medical service were significantly more likely to leave the job in the next several years and to change jobs within the profession. The two-way analysis of variance, which took into account the physicians’ place of employment and years of service, showed a significant interaction (F = 3.1, *P* = 0.027). While physicians working in health centers did not intend to leave the job regardless of years of service or their age, the intention of physicians in emergency medicine decreased with years of service (Figure 1).

### Relationship between the frequency and intensity of sources of stress and the examined responses to stress

The two groups of physicians did not always differ in the frequency and intensity of each stressor (Table 1). Therefore, the correlation analysis, instead of the index of stressfulness, took into account both frequency and intensity (Table 2). Additional reason for this was the high correlation between the index of stressfulness of specific sources of stress, which ranged from 0.502 to 0.767. It is evident that in emergency medical services the intensity of all sources of stress correlated with responses to stress, although the frequency of these stressors did not always correlate with them. Among physicians working in health centers, physical health did not depend on the frequency or intensity of conflict between work and family roles and interactions with patients, and uncontrollable working situations (frequency and intensity) did not correlate with the intent on leaving the job.

**Table 2:** Pearson correlation coefficients between assessments of frequency and intensity of sources of stress and measures of physical health, mental health, and intention of leaving the job in a convenience sample of emergency medical service  physicians (EMCI) and general practitioners working at health centers (HC)

Investigated parameters	Correlation coefficient (r) in					
	EMCI (n = 79)			HC (n = 81)		
	physical health	mental health	leaving intention	physical health	mental health	leaving intention
Uncontrollable work situations:						
frequency	0.211; *P* = 0.061	0.379; *P* = 0.001	0.149; *P* = 0.192	0.260; *P* = 0.019	0.361; *P* = 0.001	0.211; *P* = 0.058
intensity	0.269; *P* = 0.016	0.418; *P* < 0.001	0.258; *P* = 0.022	0.328; *P* = 0.003	0.346; *P* = 0.002	0.180; *P* = 0.108
Working conditions:						
frequency	0.319; *P* = 0.004	0.471; *P* < 0.001	0.230; *P* = 0.042	0.433; *P* < 0.001	0.450; *P* < 0.001	0.310; *P* = 0.005
intensity	0.319; *P* = 0.004	0.526; *P* < 0.001	0.367; *P* = 0.001	0.314; *P* = 0.004	0.397; *P* < 0.001	0.349; *P* = 0.001
Work/family conflict:						
frequency	0.460; *P* < 0.001	0.644; *P* < 0.001	0.355; *P* = 0.001	0.177; *P* = 0.115	0.460; *P* < 0.001	0.231; *P* = 0.037
intensity	0.455; *P* < 0.001	0.652; *P* < 0.001	0.366; *P* = 0.001	0.123; *P* = 0.273	0.415; *P* < 0.001	0.277; *P* = 0.012
Relationships with coworkers:						
frequency	0.309; *P* = 0.006	0.401; *P* < 0.001	0.221; *P* = 0.051	0.426; *P* < 0.001	0.434; *P* < 0.001	0.410; *P* < 0.001
intensity	0.391; *P* < 0.001	0.465; *P* < 0.001	0.300; *P* = 0.007	0.418; *P* < 0.001	0.398; *P* < 0.001	0.456; *P* < 0.001
Interaction with patients:						
frequency	0.256; *P* = 0.023	0.421; *P* < 0.001	0.189; *P* = 0.095	0.216; *P* = 0.053	0.404; *P* < 0.001	0.296; *P* = 0.007
intensity	0.335; *P* = 0.003	0.455; *P* < 0.001	0.278; *P* = 0.013	0.174; *P* = 0.121	0.319; *P* = 0.004	0.214; *P* = 0.056

## Discussion

Our study showed that the most stressful stressors in emergency medicine physicians were those from the group of uncontrollable situations and in physicians working in health centers those from the group of organization and working conditions. The stressors singled out as the strongest and most common constitute the main characteristics of the physicians’ workplaces. The work of emergency medicine physicians is characterized by unexpected work situations (8), the intensity of which is connected to all stress outcome measures. On the other hand, the work of physicians working in health centers is characterized by administrative jobs, routine procedures, compliance with medical records, and a large number of patients. Besides that, adherence to guidelines (for example minimal number of patients) and restrictions by the Ministry of Health (for example limited sources for high-quality care of patients) have additional negative effects on the organization of work and working conditions. Obtained results are in accordance with previous Croatian research (4). In both groups of physicians, the most intensive typical stressors were followed by stressors associated with interactions with patients. This is in accordance with the fact that jobs related to the care for other people are among the most stressful jobs (1-3).

Except for the organization of work and working conditions, all other sources of stress were identified as more stressful by emergency medicine physicians than by physicians working in health centers The results obtained are consistent with the initial assumption, and can be attributed to the unpredictable nature of work and specific work environment in emergency medicine (8).

Since emergency medicine physicians had worse physical health than general practitioners, it can be suggested that stressfulness of their work affected their health, primarily in terms of symptoms of psychosomatic disorders (the physical health scale, additional web material, Appendix 3). In such occupations, psychological reactions to the stressfulness of the work are often associated with burnout (20). There were no differences in mental health between the two groups of physicians, possibly due to some shared characteristics which characterize members of such professions. On the other hand, differences in physical health were probably the result of additional physical activity (12-hour shift, field work, emergency response, etc.) and the cumulative effects of increased intensity and frequency of stress in almost all emergency medicine physicians. For example, work-family conflict and interaction with patients seem to be more important contributors to impaired physical health in emergency care physicians, but not in physicians working in health centers (Table 2).

Emergency medicine physicians had a higher intention of leaving the job than physicians working in health centers, primarily being more likely to change the jobs within the profession. This might have been a result of their being younger than physicians working in health centers, and their view of emergency medical service merely as a starting point in their career. This is supported by the fact that their intention of leaving the job decreased with the years of service. Physicians working in health centers were less inclined to leave the job, regardless of years of service.

Although we only measured the intention of leaving the job, it is possible that the general fluctuation from one branch of medicine to another or even seeking for a totally different job may be explained by employment of physicians who cannot adapt to increased stress levels and/or have health problems brought about by stress (physical and mental health). While the intention of physicians working in health centers of leaving the job depended on their psycho-physical health, probably because they were older, the intention of leaving the job in emergency medicine physicians did not correlate with their psychophysical health, although they assessed their physical health as poorer. However, we should not ignore the fact that in this group, the negative features of the job are connected with the intention of leaving the job.

In conclusion, we believe that Croatian physicians need to be able to have a solid specialization in emergency medicine as opposed to the current situation when this job is performed mostly by junior physicians who soon leave the specialty because of stressful conditions.

Since this is a small pilot study, its limitations include the small number of participants and differences in some characteristics of the two samples that could have affected the results. Further studies should include the control of relevant variables that were not controlled in this study, such as differences in organizational characteristics of Emergency Medical Care Institutions and Health centers, which can represent objective measures of sources of stress. The results are correlational and do not imply causality between demographic variables and sources of stress and output stress measures. Further studies of stress in physicians should be performed longitudinally to gain deeper insight into psychophysiological health and leaving intention of Croatian physicians.
