# Supplementary material for: Stress among Croatian physicians: comparison between physicians working in emergency medical service and health centers – pilot study
Source: Croat Med J. 2011 Feb;52(1):8–15. doi: 10.3325/cmj.2011.52.8 (PMC3051263; doi:10.3325/cmj.2011.52.8)
Supplement: Supplementary Appendix 1 [file CroatMedJ_52_s001.pdf]

**Appendix 1**

**Scales of sources of stress**

The following scales include some specific sources of stress which can be related to your job. Please rate them according the **frequency of their presence** in your work and according to the **stressfulness** (amount of stress) you think they have placed on you. Please, rate frequency and stressfulness according the scales shown below.

***How often…***

**0 – never**
**1 – sometimes**
**2 - frequently**

***How much stressful ...***

**0 – not at all stressful**
**1 – mainly not stressful**
**2 – mainly stressful**
**3 – very stressful**

***UWS****

| | **How often** | **How much stressful** |
|---|---|---|
| Talking to upset patients' associates (family members, friends) | 0 1 2 | 0 1 2 3 |
| Coping with patients' death | 0 1 2 | 0 1 2 3 |
| Exposure to violent behavior and threats | 0 1 2 | 0 1 2 3 |
| The patient coping with his progressive illness whose prognosis is poor | 0 1 2 | 0 1 2 3 |
| The responsibility for patients' lives | 0 1 2 | 0 1 2 3 |
| Attendance and expectation of a call for help | 0 1 2 | 0 1 2 3 |
| Fear of infection with a contagious disease | 0 1 2 | 0 1 2 3 |
| The (non)ability to provide adequate treatment | 0 1 2 | 0 1 2 3 |
| Fear of the media and legal assessment methods, course and timing of treatment | 0 1 2 | 0 1 2 3 |
| Lack of time for punctual intervention | 0 1 2 | 0 1 2 3 |
| The unpredictability of work | 0 1 2 | 0 1 2 3 |
| Coping with atypical symptoms of known diseases | 0 1 2 | 0 1 2 3 |
| The lack of relevant data for proper making decisions about diagnosis and treatment | 0 1 2 | 0 1 2 3 |
| Night visits and interventions | 0 1 2 | 0 1 2 3 |
| Negative publicity in media | 0 1 2 | 0 1 2 3 |
| Conducting emergency response to inappropriate places and inadequate conditions | 0 1 2 | 0 1 2 3 |
| The anticipation of unexpected and urgent | 0 1 2 | 0 1 2 3 |

***OW****

| | How often | | | How much stressful | | | |
|---|---|---|---|---|---|---|---|
| Poor organization of work when there are a lot of patients in the waiting rooms | 0 | 1 | 2 | 0 | 1 | 2 | 3 |
| Time pressure and lack of time to perform the entire job | 0 | 1 | 2 | 0 | 1 | 2 | 3 |
| Impact of work on the quality of personal and family | 0 | 1 | 2 | 0 | 1 | 2 | 3 |
| A lack of the necessary work equipment and materials | 0 | 1 | 2 | 0 | 1 | 2 | 3 |
| A patient's refusal of the proposed methods of | 0 | 1 | 2 | 0 | 1 | 2 | 3 |
| The need of continuing learning | 0 | 1 | 2 | 0 | 1 | 2 | 3 |
| Performing administrative tasks | 0 | 1 | 2 | 0 | 1 | 2 | 3 |
| A great amount of responsibility | 0 | 1 | 2 | 0 | 1 | 2 | 3 |
| A sense of decreased working efficiency | 0 | 1 | 2 | 0 | 1 | 2 | 3 |
| Coping with a rare and little known disease | 0 | 1 | 2 | 0 | 1 | 2 | 3 |
| Bad working conditions | 0 | 1 | 2 | 0 | 1 | 2 | 3 |
| Prescribed financial recommendations of the ministry of health | 0 | 1 | 2 | 0 | 1 | 2 | 3 |
| Finding a replacement in a situation of having some other obligations | 0 | 1 | 2 | 0 | 1 | 2 | 3 |
| Unpredictability of work | 0 | 1 | 2 | 0 | 1 | 2 | 3 |

***W/FC****

| | How often | How much stressful |
|---|---|---|
| Working hours affect my family/personal life | 0 1 2 | 0 1 2 3 |
| Organization of working time (shift work) affects my family/personal life | 0 1 2 | 0 1 2 3 |
| Stress at work affects my communication with relatives and close persons | 0 1 2 | 0 1 2 3 |
| Lack of sleep | 0 1 2 | 0 1 2 3 |
| Lack of recognition for contributions to the profession and work | 0 1 2 | 0 1 2 3 |
| Lack of time for research | 0 1 2 | 0 1 2 3 |
| Uncertainity in decisions of other team members | 0 1 2 | 0 1 2 3 |
| Division of time on domestic / private and business | 0 1 2 | 0 1 2 3 |
| Absence of support from colleagues | 0 1 2 | 0 1 2 3 |
| Professional isolation | 0 1 2 | 0 1 2 3 |

***RC****

| | How often | How much stressful |
|---|---|---|
| Making the right decisions by itself | 0 1 2 | 0 1 2 3 |
| Fear from my own bad decisions and reactions | 0 1 2 | 0 1 2 3 |
| Problems in relationships with colleague | 0 1 2 | 0 1 2 3 |
| Inadequate treatments | 0 1 2 | 0 1 2 3 |
| Problems with managers | 0 1 2 | 0 1 2 3 |
| Coordination of decisions within the team | 0 1 2 | 0 1 2 3 |
| Conflict between my own department and other health services with which cooperation is | 0 1 2 | 0 1 2 3 |
| Conflicts with nurses and technicians | 0 1 2 | 0 1 2 3 |
| Conflicts with junior/senior fellow physicians | 0 1 2 | 0 1 2 3 |
| Anxiety from patients complaints | 0 1 2 | 0 1 2 3 |
| Unrealistic high expectancies from colleagues at [illegible] | 0 1 2 | 0 1 2 3 |

***IP****

| | **How often** | **How much stressful** |
|---|---|---|
| Demanding patients | 0 1 2 | 0 1 2 3 |
| Inappropriate demands of patients | 0 1 2 | 0 1 2 3 |
| Coping with the problems of patients which are not directly associated with the disease | 0 1 2 | 0 1 2 3 |
| Conflicts with problematic patients | 0 1 2 | 0 1 2 3 |
| Problems with premature discharge from hospital | 0 1 2 | 0 1 2 3 |
| Coping with unrealistic high expectancies from [illegible] | 0 1 2 | 0 1 2 3 |
| Anxiety about own financial status | 0 1 2 | 0 1 2 3 |
| Ingratitude of patients | 0 1 2 | 0 1 2 3 |

***Note for stressor scales titles**

UWS Uncontrollable work situations

OW The organization of work and working conditions

W/FC Conflict of work and family roles

RC Unfavorable relationships with colleagues

IP Interaction with patients
