# Supplementary material for: Stress among Croatian physicians: comparison between physicians working in emergency medical service and health centers – pilot study
Source: Croat Med J. 2011 Feb;52(1):8–15. doi: 10.3325/cmj.2011.52.8 (PMC3051263; doi:10.3325/cmj.2011.52.8)
Supplement: Supplementary Appendix 2 [file CroatMedJ_52_s002.pdf]

Appendix 2

ILJ*

This section of the questionnaire is concerned with your intentions of leaving a job. Please answer by circling the number which best represents your answer on the scale shown.

1 - Absolutely no
2 - (mostly) no
3 - No and yes ?
4 - (Mostly) yes
5 - Absolutely yes

I intent to leave this job during the next few years	1	2	3	4
I intent to change a job within	1	2	3	4
I intent to leave direct patient	1	2	3	4
I intent to leave medical work completely	1	2	3	4

*** ILJ The intentions of leaving the job**
