# Supplementary material for: Stress among Croatian physicians: comparison between physicians working in emergency medical service and health centers – pilot study
Source: Croat Med J. 2011 Feb;52(1):8–15. doi: 10.3325/cmj.2011.52.8 (PMC3051263; doi:10.3325/cmj.2011.52.8)
Supplement: Supplementary Appendix 3 [file CroatMedJ_52_s003.pdf]

Appendix 3

PS*

Please, rate how often you felt disturbances given in table below **during past year**.

Swollen joints and sore ‡	never	rarely	frequentl	always
Pain in your feet‡	never	rarely	frequentl	always
Feeling of fatigue in the morning†	never	rarely	frequentl y	always
Feeling of fatigue and exhaustion†	never	rarely	frequentl y	always
Headaches†	never	rarely	frequentl	always
Difficulty in breathing‡	never	rarely	frequentl	always
Pressure or chest pain‡	never	rarely	frequentl	always
Rapid or irregular	never	rarely	frequentl	always
Gastrointestinal	never	rarely	frequentl	always
High blood pressure	never	rarely	frequentl	always
Insomnia†	never	rarely	frequentl	always
Drowsiness†	never	rarely	frequentl	always
Poor appetite‡	never	rarely	frequentl	always

* PS **The scale of psychosomatic symptoms**

† mental health subscale

‡ physical health subscale
